# Expression of insulin-like 3 (INSL3) and differential splicing of its receptor in the ovary of rhesus macaques

**DOI:** 10.1186/1477-7827-8-150

**Published:** 2010-12-07

**Authors:** Carol B Hanna, Shan Yao, Maristela C Patta, Jeffrey T Jensen, Xuemei Wu

**Affiliations:** 1 Division of Reproductive Sciences, Oregon National Primate Research Center, Oregon Health & Science University, West Campus, Beaverton, OR 97006, USA; 2 Department of Obstetrics and Gynecology, Oregon Health & Science University, Portland, OR 97239, USA

## Abstract

**Background:**

Although insulin-like 3 (INSL3) has been identified in the gonad of both sexes in many species, there are only limited reports on the distribution of INSL3 and its receptor, relaxin/insulin-like family peptide receptor 2 (RXFP2), in the primate ovary. Since the hormone-receptor pair is believed to play a role in female reproduction, investigating the transcription of *INSL3/RXFP2 *genes and the spatiotemporal expression of INSL3 in the nonhuman primate may shed light on the functional aspects of the system in humans.

**Methods:**

Database mining, molecular and immunological methods were applied.

**Results:**

One single *INSL3 *transcript and three novel splice variant transcripts of *RXFP2 *were identified in the ovary of rhesus macaques. While the full-length *RXFP2 *transcript is barely detectable in granulosa cells during the periovulatory period, INSL3 transcript and protein are highly abundant in theca cells surrounding antral follicles. Moreover, the INSL3 level in follicular fluid is 3-4 times higher than that in female serum which remains low throughout the menstrual cycle.

**Conclusions:**

The presence of INSL3 and its receptor in the ovary implies a potential role of the ligand-receptor pair in female reproduction in nonhuman primates. However, the existence of multiple splice variants of RXFP2 indicates a very complex nature of the hormone-receptor system.

## Background

Insulin-like 3 (INSL3), also known as Leydig cell insulin-like peptide (Ley-IL) and relaxin-like factor (RLF), was originally discovered in the porcine during a search for testis-specific genes [1]. Closely related to relaxin, INSL3 peptide is a heterodimer of A and B peptides linked by disulphide bonds. In mice, deletion of INSL3 leads to bilateral intra-abdominal cryptorchidism in males due to defects in gubernacular differentiation, and reduced fertility in females due to disrupted estrous cycles [2]. Later studies also revealed that INSL3 promoted oocyte maturation independent of the mid-cycle luteinizing hormone (LH) surge in the ovary, and suppressed germ cell apoptosis in the testis in rodents [3]. Relaxin/insulin-like family peptide receptor 2 (RXFP2), also known as Leucine-rich G protein-coupled receptor 8 (LGR8) or G protein-coupled receptor affecting testis descent (GREAT), is believed to be the sole receptor for INSL3 as mice lacking RXFP2 exhibit the same testicular phenotype as that of *Insl3 *knockout mice [4,5]. Although decreased blood INSL3 levels and mutations in *Insl3/RXFP2 *genes have been linked to a small number of human cryptorchidism cases [6-10], functions of the INSL3-RXFP2 system in species other than mice are largely unknown.

Expression of the INSL3-RXFP2 system has been detected in many mammalian species including rodents, domestic and wild animals as well as primates [11-17]. The relatively high circulating INSL3 levels in male rodents and men indicate a substantial role of INSL3 as a sex-linked endocrine factor; however, the serum INSL3 levels in females are much lower [18].

In the present study we report the identification and expression of the INSL3-RXFP2 system in a nonhuman primate species, the rhesus macaque. Taking molecular and immunological approaches, we identified the macaque *INSL3 *and *RXFP2 *transcripts and splice variants in the ovary of rhesus monkeys, and investigated the tissue and cellular localization of INSL3 at both mRNA and protein levels. Due to the close resemblance of reproductive physiology between macaque species and humans, these findings may provide insights into the functional significance of the INSL3-RXFP2 signalling pathway in human female fertility.

## Methods

### Animals and tissue collection

Macaque tissues were collected from adult rhesus monkeys between 5 and 15 years of age through a tissue distribution program provided by the Division of Animal Resources (DAR) at the Oregon National Primate Research Center (ONPRC). All animal protocols and procedures were approved by the Institutional Animal Care and Use Committee (IACUC) of Oregon Health & Science University (OHSU) and were conducted in accordance with the National Institute of Health's Guide for the Care and Use of Laboratory Animals.

### Database mining, 5'-rapid amplification of cDNA ends (5'-RACE) and amplification of *RXFP2 *splice variants

Human *INSL3 *mRNA sequence (NM_005543) was used to search a rhesus macaque genome database [19] and a non-human, non-mouse expressed sequence tag (EST) database [20] for the macaque *INSL3 *homologue, and gene-specific primers (Table 1) were designed to amplify the entire *INSL3 *mRNA sequence from a monkey testis cDNA pool. Using a predicted macaque *RXFP2 *mRNA sequence (XM_001118169.1) as template, we designed a specific primer (Table 1) to amplify the 5'-end of *RXFP2 *from the macaque uterus. 5'-RACE-ready cDNA was generated following the protocol provided by the SMART RACE cDNA Amplification Kit (Clontech, Moutain View, CA, USA). The exon-intron boundaries in macaque *RXFP2 *mRNA were determined by alignment with the human *RXFP2 *mRNA sequence using Vector NTI software (Invitrogen, Carlsbad, CA, USA).

**Table 1:** Primers used for RT-PCR amplification of INSL3, RXFP2 and PPIA

Gene Name	**GenBank Acc. No**.	Primer Sequence	**T**_**A**_
*INSL3*	HM102325	RT-PCR primers:	60°C
		Forward: 5'- ATAAGTTGTGCGGCCACCAC	
		Reverse: 5'- TTTATGGTGCTGTGTGGCCTC	
*RXFP2*	GU326354	RT-PCR primers:	66°C
		Forward: 5'- TCGCTCACAGTGCTGTTTCTG	
		Reverse: 5'-AGGATATTGTTAGCCAAGAGGTCCTC	
		5'-RACE primer:	
		5'-TCCAGGTCTGAGAGTTGTGATGCGG-3'	
		Primers spanning from exons 6 to 17:	
		Forward: 5'-TGCATTAGACACATATCCAGG-3'	
		Reverse: 5'- ACTGAGGTCTGCCGTTTTC-3'	
*PPIA *(Cyclophilin A)	BC137058.1	Forward: 5'-GCTGGACCCAACACAAATG	56°C
		Reverse: 5'-TCTTCTTGCTGGTCTTGCC	

T_A_: annealing temperature

Macaque *RXFP2-*specific primers (Table 1) spanning exons 6 to 17 were designed to amplify macaque *RXFP2 *coding sequence from macaque uterus and ovary RACE-ready cDNA pools. PCR was performed for 40 cycles with Phusion high-fidelity DNA polymerase (New England Biolabs Inc., Ipswich, MA, USA) in a DNA Engine thermal cycler (Bio-Rad Laboratories, Hercules, CA, USA). The PCR amplicons were cloned into the PCR cloning vector pGEMT (Promega, Madison, WI, USA) and subjected to DNA sequencing.

### Collection of macaque oocytes, granulosa cells, and theca cells

For oocyte and granulosa cell collection, the controlled ovarian stimulation (COS) protocol, follicular aspiration, and oocyte collection procedures were performed as previously reported [21,22]. Human chorionic gonadotropin (hCG) was administered as a LH mimic to initiate periovulatory events. Aspirated follicular fluid containing granulosa cells (GC) were filtered through a 70-μm cell strainer (Becton Dickinson Labware, Franklin Lakes, NJ, USA) and precipitated at low speed. GC were recovered through low-speed centrifugation in 40% percoll/Hanks balanced salt solution (HBSS) (Invitrogen). GC obtained from antral follicles prior to hCG treatment were considered non-luteinized GC (NLGC), while those collected after hCG administration were considered luteinized GC (LGC). Theca cells were collected from frozen monkey ovarian sections using a PixCell II (Arcturus, Sunnyvale, CA, USA) laser capture microdissection system following the manufacturer's protocol.

### RNA isolation, RT-PCR, and in situ hybridization

Total RNA was extracted from monkey tissues and granulosa cells using Trizol reagent (Invitrogen), and from monkey oocytes and laser captured theca cells using Absolutely RNA Nanoprep Kit (Stratagene, La Jolla, CA, USA) following the recommended manufacturers' protocols. The integrity of total RNA was assessed using an Agilent 2100 Bioanalyzer (Agilent Technologies Inc., Santa Clara, CA, USA). Total RNA was then treated with RNase-free DNase (Promega, Madison, WI, USA) and reverse-transcribed into cDNA with Oligo (dT)_12-18 _primers and Superscript III Reverse Transcriptase (Invitrogen). RT-PCR primer pairs and corresponding annealing temperatures are listed in Table 1. PCR was performed with HotstarTaq DNA polymerase (Qiagen, Valencia, CA, USA) and all PCR products were verified by DNA sequencing. *PPIA *(PeptidylProlyl Isomerase A, also known as cyclophilin A) served as an internal control.

Selected macaque *INSL3 *and *RXFP2 *amplicons obtained by RT-PCR were cloned into the pGEMT vector (Promega). Monkey ovaries and testes were fixed in 4% paraformaldehyde, embedded in paraffin, and sectioned at 5-μm intervals. α-^35^S-UTP-labeled antisense and sense probes were generated by the Riboprobe T7/SP6 combination systems (Promega). Hybridization was carried out as previously described [23,24]. The paraffin sections hybridized with α-^35^S-UTP-labeled sense probe were used as a negative control.

### Protein extraction and Western blot

Macaque tissue total protein was extracted using 1× RIPA buffer (50 mM Tris-HCl pH7.4, 150 mM NaCl, 2 mM EDTA, 1% NP-40, 0.1% SDS) containing 1× Complete Protease Inhibitor Cocktail (Roche, Basel, Switzerland). Eighty μg of total protein in 1× Laemmli sample buffer (Bio-Rad Laboratories) was heated at 85°C for 5 minutes and then cooled to room temperature before being separated on a precast 4-20% polyacrylamide Tris-HCl gel (Bio-Rad Laboratories). The protein was transferred onto a PVDF membrane (Millipore, Billerica, MA, USA). The membrane with immobilized protein was blocked with StartingBlock T20 (TBS) Blocking Buffer (Pierce, Rockford, IL, USA) first, and then blotted with a rabbit anti-INSL3 antibody (ab65981, Abcam, Cambridge, MA, USA) at a 1:1,000 dilution. HRP-conjugated anti-rabbit IgG (Santa Cruz Biotechnology) was used as secondary antibody at a 1:10,000 dilution. Following a 5-minute incubation with ECL Western blotting substrate (Millipore), the signal was visualized on a Kodak Biomax light film (Kodak, Tokyo, Japan). The size of the detected protein band was determined by the PageRuler™ Plus Prestained Protein Ladder (Fermantas International Inc., Burlington, Ontario, Canada) separated on the same gel. The same membrane was stripped and re-blotted with HRP-conjugated mouse monoclonal beta-ACTIN (ab20272, Abcam) antibody (1:8,000 dilution), which served as an internal loading control.

### Immunohistochemistry (IHC)

Macaque ovary paraffin sections at 5-μm thickness were used for IHC. Before incubating with a rabbit anti-INSL3 antibody (H-035-48, Phoenix Pharmaceuticals, Burlingame, CA) which recognizes INSL3 precursor, the ovarian sections were deparaffinised, hydrated, and blocked with a universal blocking reagent named CAS BLOCK™(Invitrogen). IHC was performed with EnVision™dual link HRP (DAB+) kit (Dako, Carpinteria, CA, USA) according to the manufacturer's manual. The INSL3 antibody was diluted at 1:2,000, and the sections were counterstained with hematoxylin. Normal rabbit serum-stained sections were used as a negative control.

### Radioimmunoassay (RIA)

Individual macaque blood and follicular fluid samples were collected from 3-4 different monkeys and measured separately for INSL3 level with an INSL3 (human)-RIA kit (Phoenix Pharmaceuticals) following the manufacturer's protocol. The kit has a standard range of 10-1280 pg/ml with a minimum detectable range of 20.17 pg/ml. The intra- and inter-assay coefficient of variation is 5-7% and 12-15%, respectively. Female serum at follicular phase was collected from day 1 to day 14 of the menstrual cycle, while luteal phase serum was collected from day 15 to day 28 of the cycle. Follicular fluid was collected from preovulatory follicles during COS protocols [21]. Follicular aspiration was performed in animals at day 8 of COS protocols prior to hCG treatment (0 h) and at 36 hrs post hCG (36 h). The values are presented as mean ± SE (standard error).

All experiments described above were replicated at least three times with biological samples derived from different animals. Tissues and cells collected from each animal were assayed individually.

## Results

### Identification of *INSL3 *and *RXFP2 *transcripts in rhesus macaques

A full-length macaque *INSL3 *mRNA sequence without the poly(A) tail was amplified from the monkey testis cDNA pool and deposited in the GenBank database [25] with accession number HM102325. The macaque *INSL3 *homologue comprises 2 exons and shares ~93% identity with its human counterpart at the nucleotide level. The deduced macaque INSL3 precursor protein is composed of 131 amino acids which are ~90% identical with that in human INSL3 (NP_005534.2).

**Figure 1:**
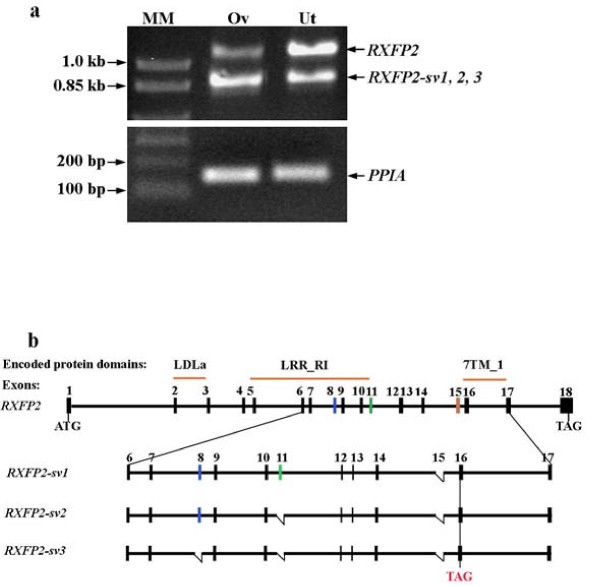
**Identification of *RXFP2 *splice variants in the ovary (Ov) and uterus (Ut)**. **a**. Agrose gel (1%) electrophoresis of PCR amplicons from both tissues. One kb Plus DNA Ladder (MM, molecular marker) (Invitrogen) was used as marker, and *PPIA *was used as an internal control. DNA was stained with ethidium bromide. The experiment was repeated with ovary and uterus cDNA derived from three monkeys; **b**. Simplified schematic representation of *RXFP2 *exons, transcripts and their corresponding encoding RXFP2 protein regions. LDLa, Low Density Lipoprotein Receptor Class A domain; LRR_RI, Leucine-rich repeats (LRRs), ribonuclease inhibitor (RI)-like subfamily; 7tm_1, 7 transmembrane receptor; ATG, translation start codon; TAG, translation stop codon.

The 5'-end of macaque *RXFP2 *mRNA was amplified by 5'-RACE with cDNA derived from the macaque uterus (GU326354), and the protein deduced from *RXFP2 *coding region shares 96% identity with human RXFP2 protein (NP_570718.1). Macaque *RXFP2 *mRNA consists of 18 exons. To demonstrate whether *RXFP2 *splice variants exist in female reproductive system of rhesus macaques, we amplified exons 6 to 17 in the ovary and uterus by RT-PCR (Figure 1a). To our surprise, besides the full-length transcript at ~1.1 kb, three distinctive splice variants were also amplified from both tissues: *RXFP2-sv1 *is 877 bp long with exon 15 missing; *RXFP2*-*sv2 *is 808 bp and lacks both exons 11 and 15; and the shortest splice variant, *RXFP2-sv3 *(738 bp), has deleted exons 8, 11 and 15 (Figure 1b). The GenBank accession numbers for three spliced transcripts are GU326355, GU326356 and GU326357, respectively.

### Distribution of *INSL3-RXFP2 *transcripts in rhesus macaques

**Figure 2:**
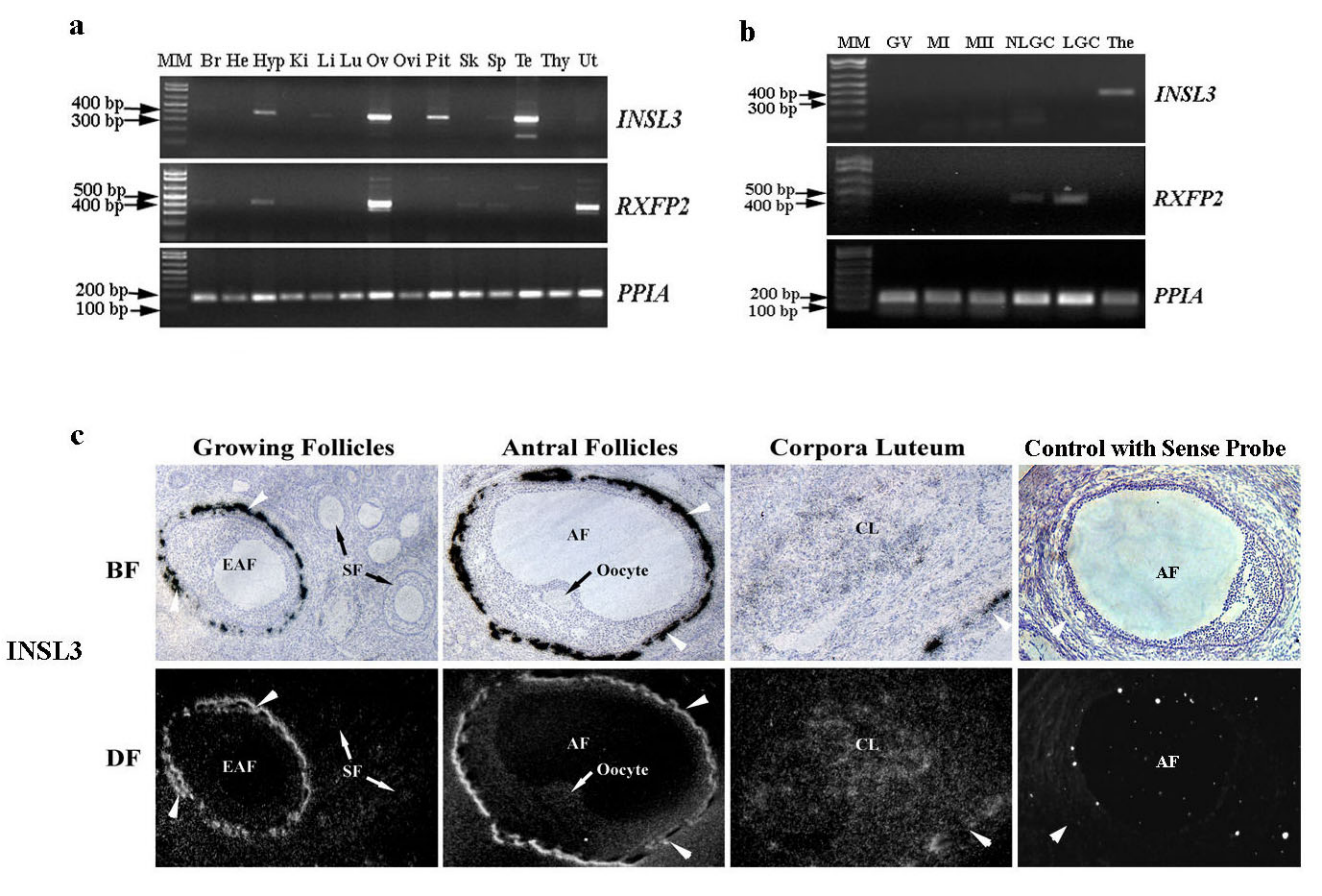
**Detection of *INSL3 *and *RXFP2 *transcripts in rhesus macaques**. **a**. Tissue distribution of *INSL3-RXFP2 *in the rhesus macaque. Total RNA extracted from the brain cortex (Br), heart (He), hypothalamus (Hyp), kidney (Ki), liver (Li), lung (Lu), ovary (Ov), oviduct (Ovi), pituitary (Pit), skeleton muscle (Sk), spleen (Sp), testis (Te), thymus (Thy) and uterus (Ut) were subjected to RT-PCR. **b**. Cellular expression of *INSL3-RXFP2 *in the macaque ovary. GV: germinal vesicle-intact oocytes; MI, MII: metaphase I, II oocytes; NLGC, LGC: non-luteinized, luteinized granulosa cells; The: Theca cells. *PPIA *was used as an internal control and 1 kb plus DNA ladder (Invitrogen) was used as molecular marker (MM) in both cases. **c**. *In situ *hybridization of *INSL3 *in the monkey ovary. White arrowheads denote theca layers of antral follicles. SF, secondary follicle; EAF: early antral follicle; AF: antral follicle; CL: corpora luteum; BF: bright field; DF: dark field. All experimental samples were collected from at least 3 individual animals.

Macaque *INSL3*-specific primers spanning both exons were used to amplify *INSL3 *transcripts from 14 macaque tissues. A cDNA fragment corresponding to 94-482 bp of macaque *INSL3 *mRNA was detectable mainly in the hypothalamus, ovary, pituitary and testis, with relatively higher abundance in the ovary and testis (Figure 2a). Macaque *RXFP2*-specific primers were designed to amplify the *RXFP2 *mRNA sequence between exons 11 and 15 so that only full-length transcript would be amplified. The full-length *RXFP2 *mRNA appears to be mainly expressed in the ovary and uterus, and weakly detectable in the hypothalamus and several other somatic tissues (Figure 2a).

The transcription of *INSL3-RXFP2 *was also examined in different cell populations in the ovary by RT-PCR (Figure 2b). Whereas *INSL3 *transcript was only detected in theca cells but not in the oocyte and granulosa cells, *RXFP2 *is expressed only in granulosa cells including both nonluteinized (NLGC) and luteinized granulosa cells (LGC). The experiment was repeated with oocytes of various stages (e.g., germinal vesicle intact, metaphase I and metaphase II) and ovarian somatic cells derived from multiple animals, and the full-length *RXFP2 *transcript was never amplified from either oocytes or thecal cells.

Consistent with the RT-PCR result, *in situ *hybridization detected abundant expression of *INSL3 *mRNA in the theca layers surrounding antral follicles, but not those surrounding secondary follicles (Figure 2c). Weak signals were also visible in corpora lutea. No signal above background was observed in either oocytes or granulosa cells. Despite RT-PCR amplification in the ovary, no specific *RXFP2 *signal was detected by *in situ *hybridization with multiple probes targeting different regions of *RXFP2 *mRNA, possibly due to a very low level expression of *RXFP2 *transcript in individual cells.

### Expression of INSL3 protein in the macaque ovary

**Figure 3:**
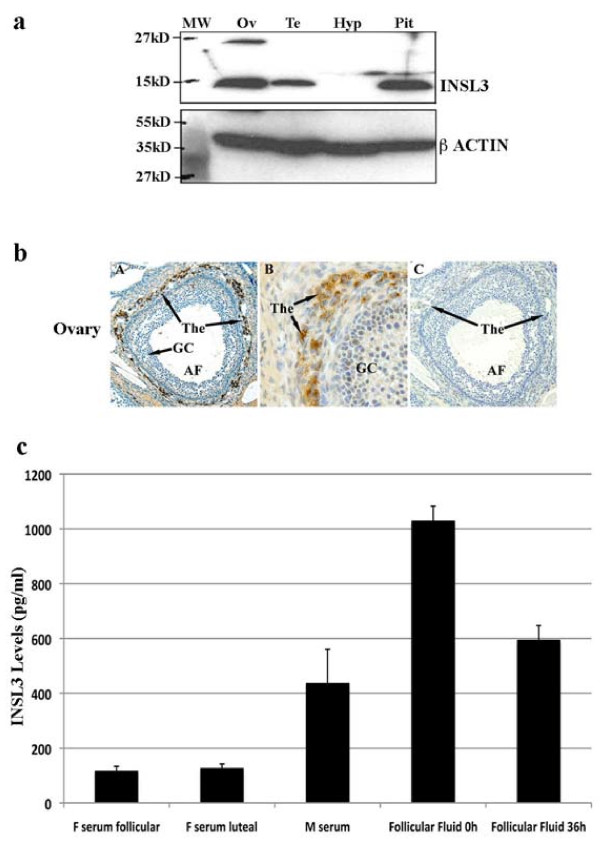
**INSL3 protein expression in selected macaque tissues**. **a**. Western blot analysis of INSL3 in selected endocrine-reproductive tissues. Eighty μg of total protein isolated from the ovary (Ov), testis (Te), hypothalamus (Hyp) and pituitary (Pit) was loaded in each lane. β-ACTIN (~47 kDa) was used as an internal loading control, and PageRuler™ Plus Prestained Protein Ladder (Fermentas) was used as molecular weight marker (MW). **b**. Immunohistochemistry (IHC) detection of INSL3 protein in the macaque ovary. Positive signal (brown) of INSL3 was localized in the thecal cells (The) surroundingantral follicles (AF), but not in other cell types within the ovary (A, B). Normal rabbit serum stained ovary section was used as negative control (C). GC, granulosa cells. **c**. INSL3 levels in monkey sera and follicular fluid during controlled ovarian stimulation (COS) protocols at 0-h and 36-h post hCG treatment. F serum follicular, female serum at follicular phase; F serum luteal, female serum at luteal phase; M, male. The value is presented as mean ± SE. Tissue lysate, sections, blood and follicular fluid were isolated from 3-4 different animals.

Using an antibody raised against human INSL3 precursor, an approximately 14.5 kDa band was detected in the macaque ovary, testis and pituitary (Figure 3a). The size of macaque INSL3 is identical to that of mouse (not shown). IHC detected specific staining of INSL3 antibody only in the theca layers surrounding antral follicles in the ovary (Figure 3b). Since only part of each macaque tissue was used for protein extraction and Western blot, quantitative analysis of the Western blot was not performed due to the uneven distribution of INSL3-expressing follicles in the ovary.

### Measurement of INSL3 levels in serum and follicular fluid in macaques

Using a highly sensitive RIA kit, we measured the INSL3 levels in adult female monkey sera during natural menstrual cycles and in follicular fluid collected during COS protocols [21]. The levels of INSL3 in the serum and follicular fluid are shown in Figure 3c. Throughout the entire menstrual cycle, there is no dramatic fluctuation of INSL3 levels in the female serum. On average, the serum INSL3 level in females (120.26 ± 7.37 pg/ml) is less than 1/3 of that in males (435.3 ± 125.6 pg/ml). However, INSL3 levels are substantially higher in the follicular fluid during the preovulatory period: 1027.78 ± 55.0 pg/ml prior to hCG treatment (0 h) and 592.59 ± 54.56 pg/ml prior to ovulation (36 h post hCG).

## Discussion

Nonhuman primates exhibit reproductive physiology that is similar to humans. For example, female rhesus macaques have 28-day mono-ovular menstrual cycles that closely resemble women's reproductive cycles, and utilize the LH-chorionic gonadotropin (CG) endocrine system unique to higher primates and absent in lower primates and nonprimates [26]. Thus, nonhuman primates are the most relevant models for studies of human reproduction. Previous studies have shown that INSL3 is more likely to have a functional endocrine role in males than in females as the blood level of INSL3 in males is significantly higher than that in females [27]. Our data demonstrates that INSL3 is also relatively enriched in the ovary of macaques, and the transcript of its full-length receptor can be detected in the ovary. The localization of the ligand to the theca layer and receptor to the granulosa cells suggests a potential local regulatory (paracrine) role of INSL3 in follicle maturation in nonhuman primates.

The structure and expression of *INSL3-RXFP2 *have been studied in primates in a limited manner. Previously an alternative splicing of *INSL3*, which possesses an extra exon (exon 1A) between exons 1 and 2, has been reported in marmoset monkeys [17] and humans (AY082014.1). Our data suggest that this particular splice variant of *INSL3 *does not exist in rhesus macaques for the primers spanning both exons consistently amplified one single transcript from different tissues derived from several monkeys. *INSL3 *mRNA has been detected in various mammalian tissues, mostly only by highly sensitive RT-PCR. In the marmoset monkey, *INSL3 *transcript was only identified in the testis by Northern blot and the ovarian corpora lutea by RT-PCR [17]. Besides the ovary and testis, we also detected *INSL3 *mRNA in the hypothalamus and pituitary in the macaque. The hypothalamic expression of *INSL3 *transcript is consistent with a previous report in the cow, but has not been observed in rodents [28]. However, an antibody recognizing INSL3 precursor failed to detect the protein in the hypothalamus despite evident INSL3 expression in the gonad and pituitary, suggesting a low-level or perhaps even no translation in this tissue. A recent report in rats demonstrated that Leydig cells in the testis could be the only source of circulating INSL3 [29]. It remains to be elucidated whether INSL3 produced in the pituitary contributes to circulating INSL3 in the rhesus macaque. It is noteworthy that the relative intensities of INSL3 bands in the Western blot described in the current study do not represent the relative abundance of INSL3 in each tissue analyzed, because the selective cell types expressing INSL3 may not distribute evenly throughout the entire tissue (e.g., theca cells surrounding antral follicles in the ovary) and only part of each macaque tissue was used for protein extraction. No specific antibodies against monkey INSL3 were available, so we tested several anti-human INSL3 antibodies and eventually selected two different antibodies for their optimal performance in Western blot and IHC. Human INSL3 shares ~90% and ~60% identical amino acids with macaque and mouse INSL3, respectively. The antibody used for Western blot was raised against a short human INSL3 peptide that only differs in 2 amino acids from mouse INSL3 precursor; thus, it is not surprising that the antibody reacts with both monkey and mouse INSL3.

Full-length macaque *RXFP2 *is transcribed in several tissues including the hypothalamus, ovary and uterus. A previous study showed that *RXFP2 *was expressed in the testis but not in the ovary in humans [30]. The contradiction could be, at least in part, attributed to the selection of primers, i.e., while only full-length *RXFP2 *transcript was amplified by primers spanning between exons 11 and 15 in our experiment, the amplification of 3'-UTR region of human *RXFP2 *would include various spliced transcripts. Indeed, the identification of three *RXFP2 *splice variants was previously reported in the human uterus and adrenal glands [31], although only one of the sequences, namely *LGR8.1 *which lacks exon 11 of *RXFP2*, can be found in the GenBank (AY899851.1). None of the three splice variants identified from the macaque corresponds exactly to *LGR8.1*, although two of them (i.e., *RXFP2-sv2 *and *RXFP2-sv3*) are missing exon 11. We noticed that the primers used to amplify *LGR8.1 *span exons 7-13 of human *RXFP2*, which could explain why the primer pair failed to identify the missing exon 15 that was found in all three splice variants reported in the current study. Unlike *LGR8.1 *that can be translated into a truncated RXFP2 protein with partial leucine-rich repeat (LRR) region, translation of all three *RXFP2 *splice variants identified in the current study can cause early termination of the full-length open reading frame (ORF) prior to the seven-transmembrane (7TM) domain-coding region (Figure 1b).

Within the ovary, INSL3 transcript and peptide are predominantly expressed in the theca cells surrounding antral follicles and weakly detectable in the corpus luteum. This finding is similar to previous observations in the cow [28] as well as in two other primate species, marmoset monkeys and humans [16,17]. INSL3 was originally demonstrated as a male hormone required for testis positioning during fetal development [2,32,33]. A previous finding showing that INSL3 was expressed in the ruminant ovary at a much higher level than other species was attributed to the natural loss of the gene for relaxin [34]. Here we show that INSL3 is also highly expressed in the primate ovary, even though relaxin gene still exists in this species [35]. The function of INSL3 in the female reproductive system has not been well characterized. Besides the potential anti-apoptotic role of INSL3 in the mouse ovary [36], Hsueh's group [3] reported that INSL3 could function as a local mediator for the mid-cycle LH surge and promote the oocyte meiotic resumption through binding to RXFP2, an inhibitory G (G_i_) protein-coupled receptor (GPCR). Although some recent evidence shows that the oocyte maturation in response to LH may be independent of G_i _proteins [37], the highly enriched local INSL3 and the presence of its receptor imply a possible local regulatory role of the ligand-receptor in the ovary, especially during preovulatory period. Notably, we did not detect *RXFP2 *transcript or protein in the germinal vesicle intact oocyte, so any effect of INSL3 on maturation must be mediated through the cumulus granulosa cells. More functional studies in higher species will be required to solve this puzzle. The failure of RXFP2 detection in the ovary could be due to very low-level expression of GPCRs in individual cells, the specificity of antibodies applied, and/or an unknown nature of *RXFP2 *gene transcription and translation. The presence of three differentially spliced transcripts of *RXFP2 *in the ovary, and the localization of full-length *RXFP2 *mRNA in granulosa cells has not been previously reported. The majority of primate genes produce splice variant transcripts [38]; it is impossible to predict whether these splice variants can actually be translated into proteins, whether these proteins are functional, or whether these alternatively spliced transcripts have a function independent of protein synthesis. However, since the reported splice variants of *RXFP2 *encode truncated protein isoforms without the seven-transmembrane domain, these protein products are unlikely to function as GPCRs even if they do exist [39,40]. It also remains to be determined whether the cellular localization of full-length *RXFP2 *transcript in the ovary is specific to rhesus monkeys.

## Conclusions

Taken together, we identified the *INSL3-RXFP2 *transcripts and determined the tissue and cellular distribution of INSL3 in the ovary of rhesus macaque monkeys. Despite the rapid increasing number of studies on this newly identified ligand-receptor pair, the expression and function of the system appear much more complex than originally thought, especially in more advanced species. Further biochemical and pharmacological studies in nonhuman primates may eventually reveal the roles of INSL3 and the significance of multiple splice variants of *RXFP2 *in the ovary.

## Competing interests

The authors declare that they have no competing interests.

## Authors' contributions

CBH carried out the identification of *INSL3 *and *RXFP2 *transcripts and splice variants, as well as PCR amplification of *INSL3 *from multiple macaque tissues. SY performed Western blot and IHC for INSL3. MCP participated in sample preparation and collection. JTJ conducted the measurement and analysis of INSL3 in the follicular fluid and blood. XW carried out INSL3 and RXFP2 sequence alignment, analysis, and manuscript preparation. All authors read and approved the final manuscript.
